# Evidence for increasing densities and geographic ranges of tick species of public health significance other than *Ixodes scapularis* in Québec, Canada

**DOI:** 10.1371/journal.pone.0201924

**Published:** 2018-08-22

**Authors:** Salima Gasmi, Catherine Bouchard, Nicholas H. Ogden, Ariane Adam-Poupart, Yann Pelcat, Erin E. Rees, François Milord, Patrick A. Leighton, Robbin L. Lindsay, Jules K. Koffi, Karine Thivierge

**Affiliations:** 1 Policy Integration and Zoonoses Division, Centre for Food-borne, Environmental & Zoonotic Infectious Diseases, Public Health Agency of Canada, Saint-Hyacinthe, Québec, Canada; 2 Groupe de Recherche en Épidémiologie des Zoonoses et Santé Publique (GREZOSP), Saint-Hyacinthe, Québec, Canada; 3 Public Health Risk Sciences Division, National Microbiology Laboratory, Public Health Agency of Canada, Saint-Hyacinthe, Québec, Canada; 4 Direction des risques biologiques et de la santé au travail, Institut national de santé publique du Québec, Montréal, Québec, Canada; 5 Faculty of Veterinary Medicine, University of Montreal, Saint-Hyacinthe, Québec, Canada; 6 Zoonotic Diseases and Special Pathogens, National Microbiology Laboratory, Public Health Agency of Canada, Winnipeg, Manitoba, Canada; 7 Laboratoire de santé publique du Québec, Institut national de santé publique du Québec, Sainte-Anne-de-Bellevue, Québec, Canada; 8 Institute of Parasitology, Faculty of Agricultural and Environmental Sciences, McGill University, Macdonald Campus, Sainte-Anne-de-Bellevue, Québec, Canada; University of Minnesota, UNITED STATES

## Abstract

Climate change is driving emergence and establishment of *Ixodes scapularis*, the main vector of Lyme disease in Québec, Canada. As for the black-legged tick, *I*. *scapularis* Say, global warming may also favor northward expansion of other species of medically important ticks. The aims of this study were to determine (1) current diversity and abundance of ticks of public health significance other than *I*. *scapularis*, (2) sex and age of the human population bitten by these ticks (3), and the seasonal and geographic pattern of their occurrence. From 2007 to 2015, twelve tick species other than *I*. *scapularis* were submitted in the Québec passive tick surveillance program. Of these 9243 ticks, 91.2% were *Ixodes cookei*, 4.1% were *Dermacentor variabilis*, 4.0% were *Rhipicephalus sanguineus* and 0.7% were *Amblyomma americanum*. The combined annual proportion of submitted *I*. *cookei*, *D*. *variabilis*, *R*. *sanguineus* and *A*. *americanum* ticks in passive surveillance rose from 6.1% in 2007 to 16.0% in 2015 and an annual growing trend was observed for each tick species. The number of municipalities where *I*. *cookei* ticks were acquired rose from 104 to 197 during the same period. Of the 862 people bitten by these ticks, 43.3% were *I*. *cookei* ticks removed from children aged < 10 years. These findings demonstrate the need for surveillance of all the tick species of medical importance in Québec, particularly because climate may increase their abundance and geographic ranges, increasing the risk to the public of the diseases they transmit.

## Introduction

Ticks are hematophagous arthropods that parasitize nearly every class of vertebrates including man in almost all parts of the world [1, 2]. Amongst the circa 900 known species of ticks, many are vectors of pathogens of importance for human and/or animal health. Ticks are considered to be second only to mosquitoes as vectors of infectious diseases of importance for human health. Each tick species has preferred environmental conditions and host species that determine their ecological niche and geographic distribution and, consequently, the distribution of risk areas for any diseases they may transmit. Anthropogenic, climate and landscape changes have, in some cases, been shown to contribute to the geographic range expansion of ticks and tick-borne diseases [3, 4]. The northward expansion of *I*. *scapularis*, the main vector of *Borrelia burgdorferi*, the agent of Lyme disease in northeastern North America, is now well documented in Canada [5, 6]. Between 2009 and 2012, the number of locations where *I*. *scapularis* ticks have been found has increased substantially in southern central and eastern Canada [7]. Leighton et al. (2012) produced model projections suggesting that the geographic range of *I*. *scapularis* will expand at a speed of 33 to 55 km per year in the coming decade [8], with climate warming expected to increase the rate of spread, and this estimate has recently been validated in the field [9]. This expansion will likely result in a substantial increase in risk of human Lyme disease, and of other diseases transmitted by *I*. *scapularis* including babesiosis, anaplasmosis, Powassan virus encephalitis, and *B*. *miyamotoi* infection [8, 10–12]. Global warming may also allow northward expansion of the geographic range of other medically important species of ticks and, consequently, emergence of public health risks from tick-borne diseases in addition to those transmitted by *I*. *scapularis*. This trend has been observed on the East Coast of the United States [13] and in the Canadian prairies [14] with the expansion of *Dermacentor variabilis* and *Dermacentor andersoni*, respectively.

The passive tick surveillance system run by the Laboratoire de santé publique du Québec (LSPQ) since 1990 mainly focuses on *I*. *scapularis* surveillance. In this surveillance system, ticks collected from human patients and their pets are submitted by participating medical hospitals and veterinary clinics to the LSPQ. However, most doctors and veterinarians participating in this program do not have the expertise to differentiate *I*. *scapularis* from other tick species, so almost half of the ticks submitted through the passive blacklegged tick surveillance system are not *I*. *scapularis*. Among these different species, many have well-known medical importance including *Ixodes cookei* (as a vector of Powassan encephalitis), *Dermacentor variabilis* [as a vector of Rocky Mountain spotted fever (RMSF) and tularaemia], *Rhipicephalus sanguineus* (a vector of RMSF) and *Amblyomma americanum* [a vector of human monocytic ehrlichiosis, human ehrlichiosis associated with *Ehrlichia ewingii* [15] and tularaemia]. Also, *A*. *americanum* is suspected to play a role in development of red meat food allergy in humans [16]. This study presents the data collected on ticks other than *I*. *scapularis* submitted through passive surveillance for ticks in Québec from 2007 to 2015, which, to date, have not been explored. The main objectives were to determine (1) the current diversity and abundance of ticks of public health significance other than *I*. *scapularis*, (2) sex and age of human population bitten by those ticks (3) and, the seasonal and geographic pattern of tick distribution.

## Materials and methods

### Tick surveillance data

For the present study, ticks other than *I*. *scapularis* collected in passive surveillance for ticks, between 2007 and 2015 were analyzed. The populations of interest in this study were i) ticks other than *I*. *scapularis* that were collected through passive surveillance and ii) the human population in Quebec bitten by tick species of public health significance.

Passive surveillance for blacklegged ticks has been ongoing in Québec since 1990. In this surveillance system, ticks collected from human patients and their pets are submitted by participating medical hospitals and veterinary clinics to the LSPQ for identification at the species level using standard taxonomic keys. Information on tick instar (larvae, nymph, adult [male or female]), tick engorgement level and travel history of the patient or pet (at municipality level) and the date of exposure are also recorded. For this study, we analyzed ticks collected from 2007 to 2015. Prior to 2009, ticks were submitted from medical and veterinary clinics across the province. However in 2009, it was decided that submissions from participating veterinary clinic located in the Montérégie health unit would no longer be accepted. This change in policy was adopted following the implementation of active tick surveillance in Montérégie in 2007. Active field surveillance involves collecting ticks directly from the environment using drag sampling (to collect questing ticks) or capture of rodent hosts (to collect feeding ticks) [7]. The passive surveillance data collected on non-human animals from the LSPQ being mainly used by epidemiologists to determine where the active surveillance should be deployed, the passive surveillance in this health unit was stopped to relocate human and financial resources in active surveillance. The collection, use, analysis and disclosure of data described in the current article fall within the surveillance mandate. Therefore, research ethics committee approval was not required.

### Descriptive analyses

Descriptive analysis of ticks’ data collected during surveillance was performed to assess (1) the diversity and abundance of the tick species and how these have changed over time, (2) the demographic characteristics of the human population bitten by ticks (from human passive surveillance data), and (3) seasonal and geographic distributions of the tick species other than *I*. *scapularis*.

Ticks collected through passive surveillance program from human and animal sources that had travelled outside their province of residence (Québec) within a 2 week period before submission of ticks were excluded from all analyses.

For the demographic analysis of the human population bitten by ticks other than *I*. *scapularis*, the proportions of submissions according to age group and sex were calculated as the incidence of tick bites per 100,000 person-year. The denominators were population sizes by age-group and sex were obtained from the Institut de la statistique du Québec (http://www.stat.gouv.qc.ca/statistiques/population-demographie/structure/index.html).

For geographic analyses using passive surveillance data, ticks collected in passive surveillance from human and animal sources that had travelled from their location of residence within a 2 week period before submission of ticks were excluded from geographic analysis. The geolocation of submitted ticks was the centroid of the CSD of submission. The number of ticks were normalized for the human population. Rates of tick submissions per 100,000 person-year were generated by using 2011 census data at the CSD level. All maps were created using Environmental Systems Research Institute’s ArcGIS v10.5 software (Redlands, California).

## Results

### Descriptive analysis of ticks submitted in passive surveillance

Between 2007 and 2015, 29,394 ticks removed from domestic animals and humans were submitted through the passive surveillance program (Table 1). Ticks species other than *I*. *scapularis* represented 43.5% of overall tick submissions (12,784/29,394). Among these ticks, 75.6% (9,664/12,784) were reported as having been acquired in Québec, 9.7% (940/9,664) were removed from humans and the remaining from animals. Of the ticks removed from animals, the majority (96.3%) were removed from dogs, cats and ferrets (51.7%, 44.5% and 3.1%, respectively). Rabbits, horses, skunks, birds and rodents each were the hosts for less than 0.5% of the ticks. Among the 12 tick species other than *I*. *scapularis* collected in passive surveillance, were *I*. *cookei*, *D*. *variabilis*, *R*. *sanguineus* and *A*. *americanum* representing 95.6% (9,243/9,664) of non-*I*. *scapularis* ticks acquired in Quebec. Of these, *I*. *cookei* was the species most frequently submitted, representing 91.1% of non-*I*. *scapularis* tick submissions, followed by *D*. *variabilis*, *R*. *sanguineus* and *A*. *americanum* (4.1%, 4.0% and 0.7%, respectively).

**Table 1:** Ticks of species other than *I*. *scapularis* collected in Québec via passive surveillance (2007–2015). The development stage (larvae, nymphs or adults) and origin of the tick specimens (human or animal) are shown for each species. Only ticks submitted from humans or animals with no travel history outside Québec two weeks before submission are reported.

Tick species	No. of ticks from humans			No. of ticks from animals			
	Larvae	Nymphs	Adults	Larvae	Nymphs	Adults	Total
*Ixodes cookei*	23	464	272	3060	2663	1945	**8427**
*Ixodes muris*	1	0	13	0	1	100	**115**
*Ixodes marxi*	0	0	15	0	0	62	**77**
*Ixodes angustus*	0	0	0	0	0	3	**3**
*Dermacentor andersoni*	0	0	0	0	0	1	**1**
*Dermacentor albipictus*	0	0	13	0	41	119	**173**
*Dermacentor variabilis*	0	0	114	0	3	265	**382**
*Rhipicephalus sanguineus*	0	0	2	3	39	327	**371**
*Amblyomma americanum*	0	3	19	0	0	41	**63**
*Amblyomma maculatum*	0	0	0	0	0	4	**4**
*Amblyomma Cajennense*	0	0	1	0	0	0	**1**
*Haemaphysalis leporispalustris*	0	0	0	18	19	10	**47**

Of the 8427 *I*. *cookei* ticks collected, 91% of them were from animals, 73.7% were immature (2217 adults, 3127 nymphs and 3083 larvae) (Table 1), and 62.4% were part of submissions of multiple ticks of the same species (Table 2). A total of 371 *R*. *sanguineus* ticks were submitted in passive surveillance, mostly from animals and most were adults (329 adults, 39 nymphs and 3 larvae), (Table 1), and 69.8% of these ticks were part of submissions of multiple ticks of the same species (Table 2). For *D*. *variabilis*, 382 specimens were collected (379 adults and 3 nymphs) amongst which 29.8% were submitted from humans (Table 1). Also, 22.2% of the overall collected *D*. *variabilis* ticks were part of submissions of multiple ticks of the same species (Table 2). *Amblyomma americanum* was less frequently submitted with 63 specimens (60 adults and 3 nymphs) mostly collected from animals (Table 1) as single submissions (Table 2).

**Table 2:** Abundance of the four tick species that were most highly submitted from humans and animals in the passive surveillance program in Québec, from 2007 to 2015: Number of *I*. *cookei*, *D*. *variabilis*, *A*. *americanum* and *R*. *sanguineus*. Results are shown by the development stage as well as by single or multiple tick submission (i.e. more than one tick collected from the same person or animal at the same time).

Tick species	Male	Female	Nymphs	Larvae	Total
*Ixodes cookei*					
Single	12	1724	1320	113	**3169**
Multiple	4	477	1807	2970	**5258**
**Total**	**16**	**2201**	**3127**	**3083**	**8427**
*Dermacentor variabilis*					
Single	94	202	1	0	**297**
Multiple	32	51	2	0	**85**
**Total**	**126**	**253**	**3**	**0**	**382**
*Amblyomma americanum*					
Single	10	48	1	0	**59**
Multiple	0	2	2	0	**4**
**Total**	**10**	**50**	**3**	**0**	**63**
*Rhipicephalus sanguineus*					
Single	28	71	10	3	**112**
Multiple	125	105	29	0	**259**
**Total**	**153**	**176**	**39**	**3**	**371**

**Fig 1:**
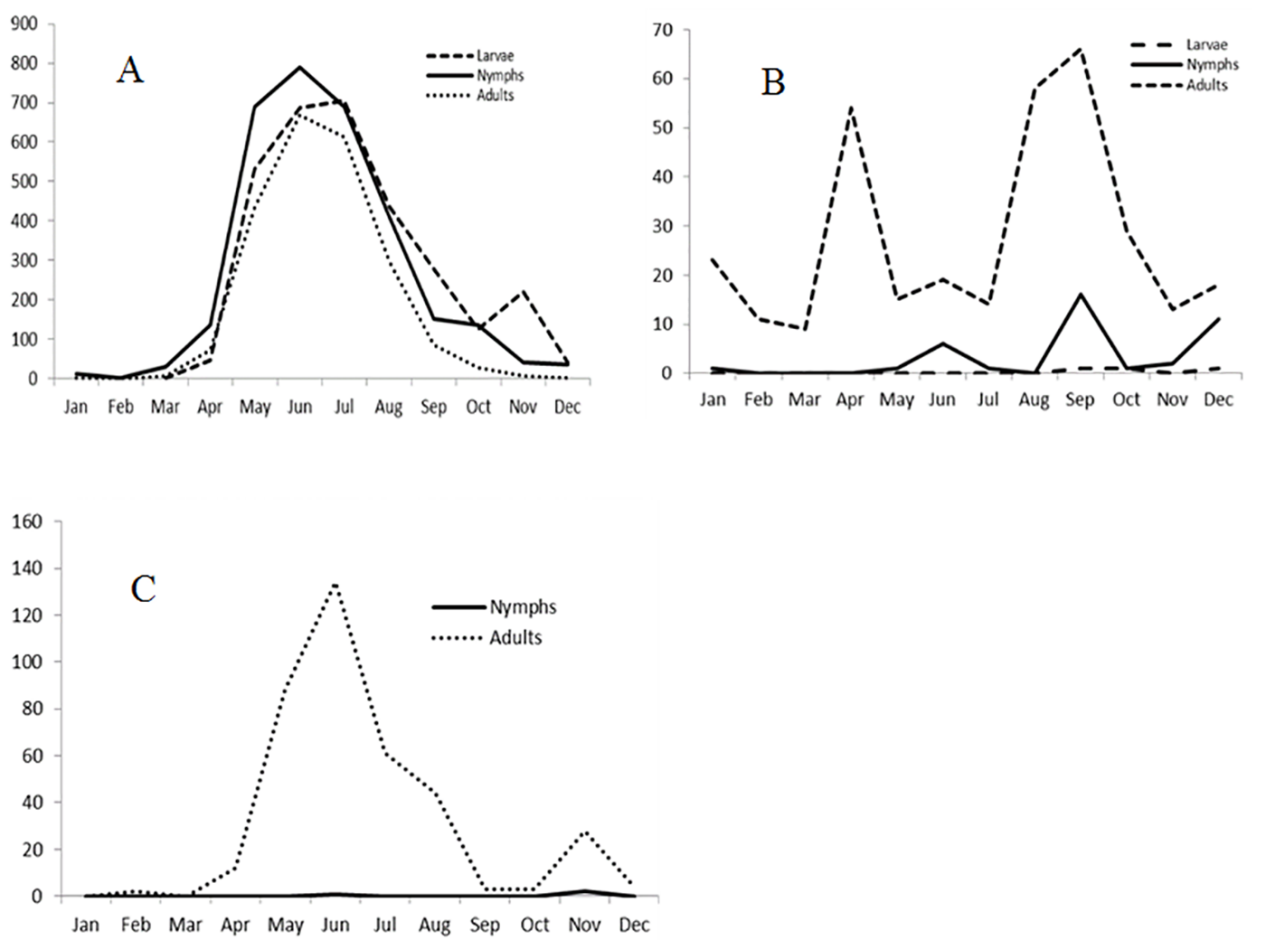
Monthly number of immature and mature ticks collected in passive surveillance in Québec from 2007 to 2015 for (A) *I*. *cookei*, (B) *R*. *sanguineus* and (C) *D*. *variabilis* ticks.

The number of submitted *A*. *americanum* was too low to describe seasonality. All instars of *I*. *cookei* ticks were submitted in April through September, with unimodal peaks of abundance of nymphs and adults occurring in June, and bimodal peaks of larval abundance in spring (slightly later than nymphs and adults) and November (Fig 1A). *Rhipicephalus sanguineus* ticks were collected during all the months of the year, with adult tick abundance peaking in April, June and September and nymph abundance peaking in June and September (Fig 1B). Adult *D*. *variabilis* ticks (Fig 1C) were collected from February to December but with most being submitted in late spring and in the summer with tick abundance peaking in June, and lowest in November.

**Fig 2:**
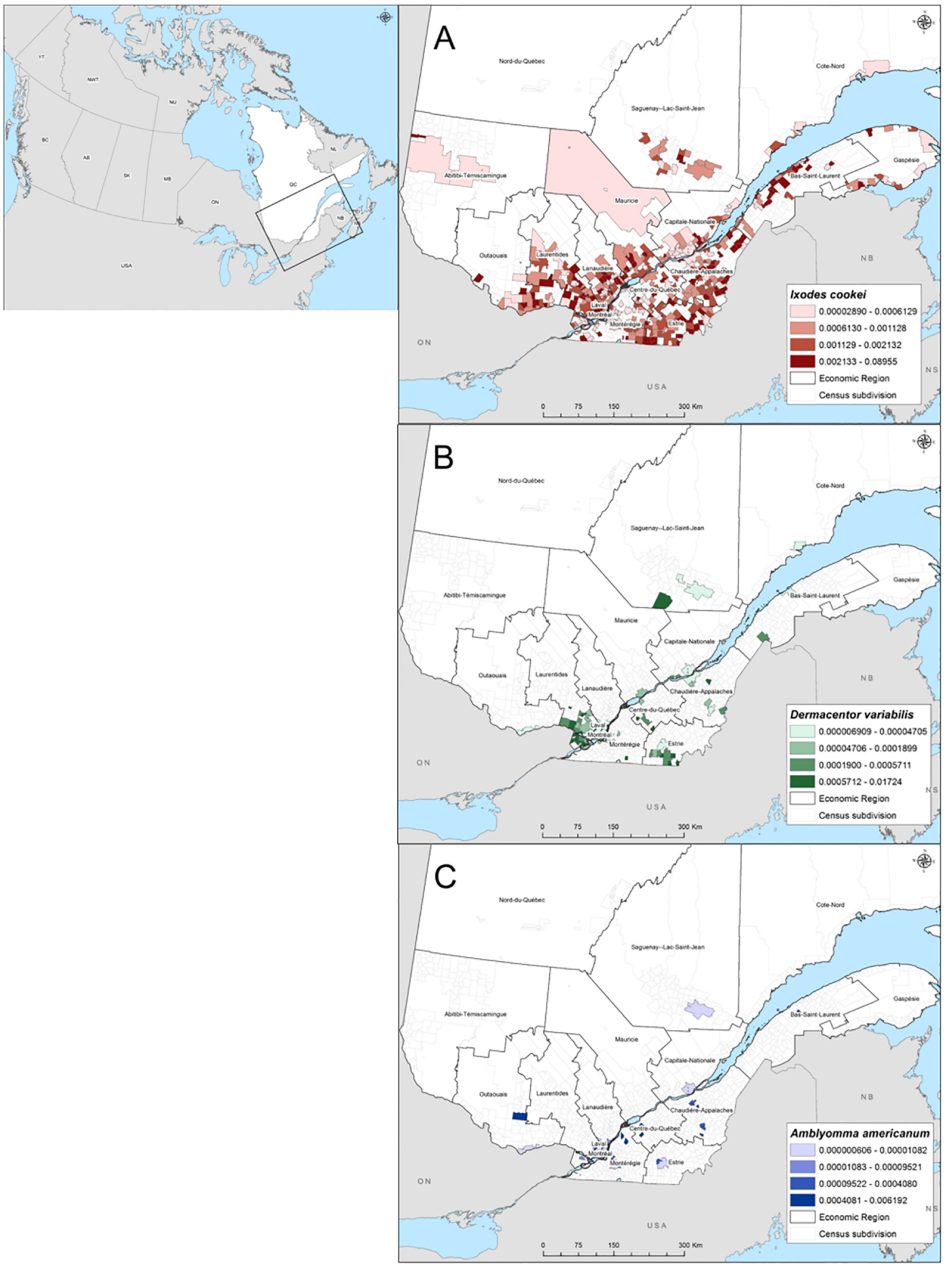
Geographic occurrence index of three tick species most highly submitted in the passive surveillance system program in Québec from 2007–2015, for (A) *I*. *cookei*, (B) *D*. *variabilis* and (C) *A*. *americanum* ticks submitted. The index is the number of submissions normalized by the human population size at the census subdivision level. Index values are grouped by quartiles.

Fig 2A shows the geographic distribution of submissions of *I*. *cookei* in Québec (n = 7138 ticks, including 1801 adults, 2552 nymphs and 2785 larvae). Submissions of this species were particularly abundant in the Estrie, Chaudière-Appalaches, Centre-du-Québec, Laval and Montreal regions. The areas along the St. Lawrence River in the Capitale-Nationale, Mauricie, Lanaudière and Laurentides regions were also high-submitting areas (Fig 2A). The low number of submissions in Montérégie demonstrates the possible bias created by the cessation of passive surveillance of ticks collected from animals in this region in 2009. Fig 2B shows the geographical distribution of *D*. *variabilis* in Québec (n = 266 ticks submitted, 263 adults and 3 nymphs). Submissions of this species were highest in the regions of Estrie, Chaudière-Appalaches, Centre-du-Québec, Saguenay-Lac-St-Jean, Laval, Montreal and the Laurentides. As for *I*. *cookei*, the low number of ticks submitted in Montérégie likely demonstrates the bias created by the cessation of passive surveillance in this region. As illustrated in Fig 2C, only a small number of ticks were available to map the geographic distribution of *A*. *americanum* (n = 41 ticks, including 38 adults and 3 nymphs), which were submitted from the regions of Bas-Saint-Laurent and the northern part of the Montérégie.

A trend of annually increasing abundance was observed for the four most frequently submitted non-*I*. *scapularis* tick species (*I*. *cookei*, *D*. *variabilis*, *R*. *sanguineus and A*. *americanum*) (Table 3). The annual proportion of submitted ticks, which were of these species, rose from 6.1% in 2007 to 16.0% in 2015. This annual trend is mainly due to increasing submissions of *I*. *cookei* ticks, which rose from 6.4% of submitted ticks in 2007 to 16.3% in 2015 as well as the number of CSDs where those ticks were acquired (from 104 in 2007 to 197 in 2015). For the other tick species, the annual proportion of submitted ticks increased 3 to 4 fold in the same period although for these species there was much more inter-annual variation in the numbers of ticks submitted (Table 3).

**Table 3:** The annual number and proportion of the four tick species that were most highly submitted from humans and animals in passive surveillance program in Québec from 2007 to 2015.

Tick species	2007	2008	2009	2010	2011	2012	2013	2014	2015	Total
*Ixodes cookei*	538 (6.4)	462 (5.5)	787 (9.3)	933 (11.1)	1069 (12.7)	975 (11.6)	933 (11.1)	1357 (16.1)	1373 (16.3)	8427 (100)
*Dermacentor variabilis*	18 (4.7)	14 (3.7)	18 (4.7)	43 (11.3)	23 (6.0)	91 (23.8)	67 (17.5)	44 (11.5)	64 (16.8)	382 (100)
*Rhipicephalus sanguineus*	8 (2.2)	40 (10.8)	25 (6.7)	86 (23.2)	36 (9.7)	54 (14.6)	62 (16.7)	24 (6.5)	36 (9.7)	371 (100)
*Amblyomma americanum*	3 (4.4)	2 (2.9)	3 (4.4)	5 (7.4)	4 (6.3)	18 (26.5)	10 (14.7)	8 (11.8)	10 (14.7)	63 (100)
**Total**	**567 (6.1)**	**518 (5.6)**	**833 (9.0)**	**1067 (11.5)**	**1132 (12.2)**	**1138 (12.3)**	**1072 (11.6)**	**1433 (15.5)**	**1483 (16.0)**	**9243 (100)**

**Fig 3:**
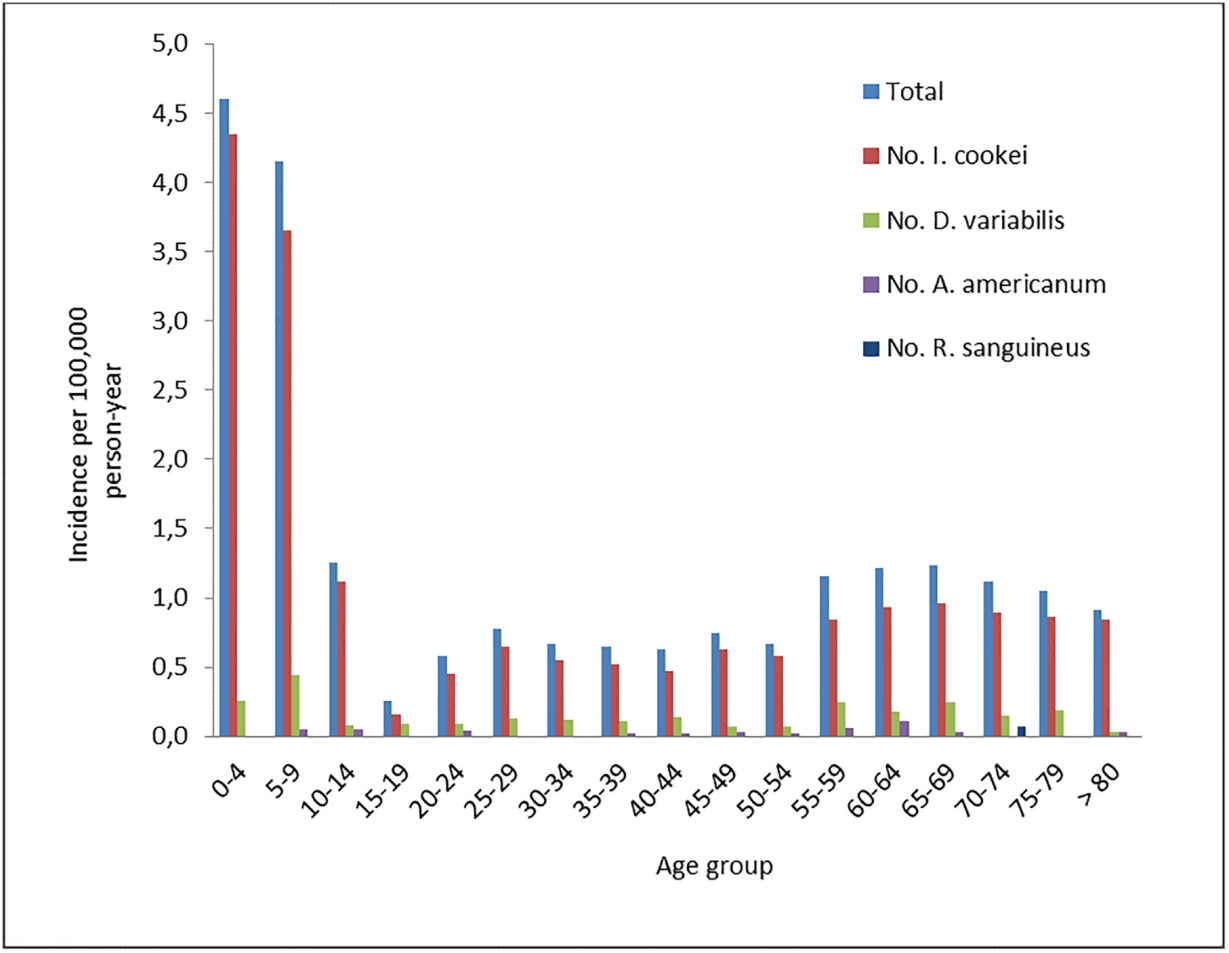
Incidence per 100,000 person-year, given age category, for tick submissions of *I*. *cookei*, *A*. *americanum*, *D*. *variabilis* and *R*. *sanguineus* (and their total) from the passive surveillance program in Québec from 2007 to 2015.

Between 2007 and 2015, 1812 ticks other than *I*. *scapularis* were collected from 1688 humans and submitted to LSPQ. Among these submitters, and for whom the data on travel region and age were available, 862 reported having acquired the four species most frequently submitted in Québec. The distribution of incidence by age group of the four species per 100,000 person-year was bimodal with peaks in the 0–9 and 55–69 age-groups (Fig 3). The majority (84.8%) of the people bitten by the four species most frequently submitted (n = 862) were bitten by *I*. *cookei* (n = 731) and consequently the distribution pattern of the incidence by age-group per 100,000 person-year was similar to that of the *I*. *cookei* age distribution (Fig 3) except for age-groups between 55 and 69 for which the incidence varied slightly between 0.8 and 1.0. Of these *I*. *cookei* tick submissions, children below 10 years of age reported the highest proportion (43.3%) and the 15–19 age group reported the lowest proportion (0.9%) of the tick-bitten population (Fig 3). For *D*. *variabilis*, the incidence peaked in children aged 5–9 years (0.4 per 100 000 person-year) and the proportion of overall incidence was highest (26.6%) in children aged below 10 years (Fig 3).

**Fig 4:**
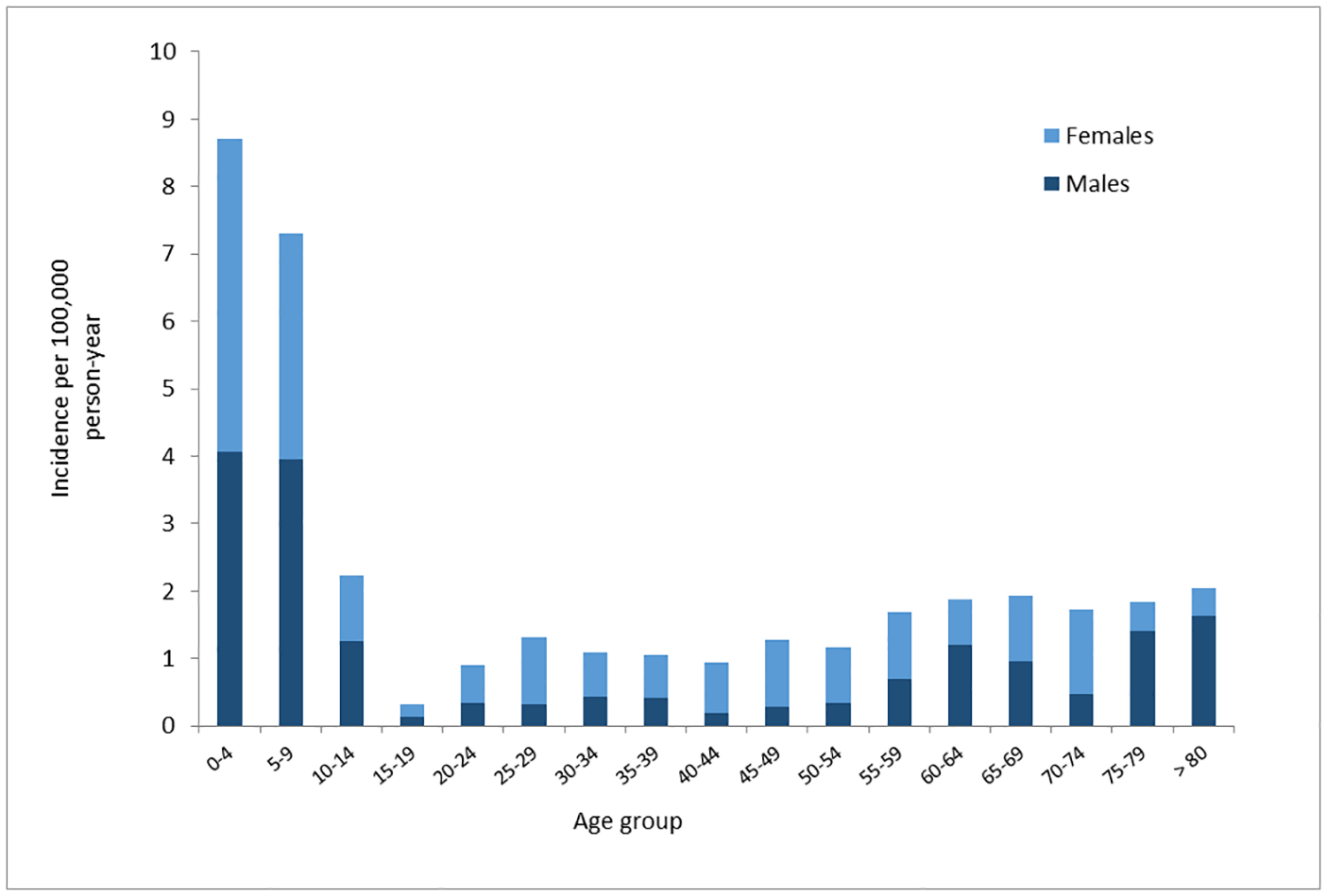
Incidence per 100,000 person-year, given age category, of *I*. *cookei* ticks submitted from the passive surveillance program in Québec from 2007 to 2015. Also shown are the proportions by sex.

For the people submitting *I*. *cookei* ticks (n = 731), females (53.8%) were more bitten than males and the incidence of *I*. *cookei* tick submission per 100,000 person-year was higher in females than males for each age-group from 15 to 59 years (the average incidence was 1.3 in females and 0.4 in males) (Fig 4).

## Discussion

Previous studies have reported on the occurrence of *I*. *scapularis* as being the most abundant tick species detected through passive and active surveillance programs in Quebec [7, 11, 17]. Here we focus on the other tick species found during the surveillance years 2007 to 2015. Our study identified twelve tick species other than *I*. *scapularis* detected in Québec. This is in contrast to seventeen tick species (excluding *I*. *scapularis*) reported in Ontario between 2008 and 2012 [18].

In Québec, the four most submitted non-*I*. *scapularis* tick species of public health significance were *I*. *cookei*, *D*. *variabilis*, *R*. *sanguineus* and *A*. *americanum*. *Ixodes cookei* was the most abundant tick species after *I*. *scapularis* in passive surveillance as reported by a recent study [17]. *Ixodes cookei* was found to be the third most frequently submitted tick after *I*. *scapularis* and *D*. *variabilis* in neighbouring Canadian province in Ontario [18] and American state of Maine [19]. These differences in diversity and abundance of tick species found between Québec and Ontario, two neighbouring regions, are likely related to differences in micro climate, host density and/or habitat [20, 21].

In Canada, *I*. *cookei* is primarily associated with the woodchuck, and has been recorded in every province from Newfoundland to southeastern Manitoba [22]. The high proportion of submitted immatures ticks of this species (approximately 3/4 of submitted specimens) and the large number of multiple tick submissions of *I*. *cookei* ticks strongly suggests that this tick is established widely in the province. *I*. *cookei* ticks were collected mostly from domestic animals (70% of submitted *I*. *cookei* ticks; max. of 140 larvae/animal and 39 nymphs/animal) and this is likely due to the potential of dogs and other pets to collect and acquire ticks in the environment and the endophilic characteristic of this tick species which remains hidden close to their host’s nest or burrows and only attach to the host when it arrives [23].

*Ixodes cookei* tick is an inefficient vector of the bacterial cause of Lyme borreliosis [24, 25] but is the primary vector of Powassan virus. This pathological agent causes a rare but severe illness [26] which is increasingly reported in the USA in States neighbouring Canada in general, and Québec in particular [27]. Children are more frequently infected with Powassan virus disease than adults in North America. Unlike *Borrelia burgdorferi*, for which *I*. *scapularis* must feed for 36 hours for transmission to occur [28], there is no such delay for *I*. *cookei* to transmit Powassan virus [29]. Currently, no vaccine or specific treatments are available to prevent and treat Powassan virus infection. Prevention is achieved by avoiding the *I*. *cookei* tick-bites by personal protective measures or avoiding areas at risk. To date, no molecular analyses have been conducted to identify the presence, and assess the prevalence of infection, of pathogens in non-*I*. *scapularis* tick species in Quebec. This limits our capacity to quantify the risk to the public from pathogens transmitted by ticks of species other than *I*. *scapularis*. However, although *I*. *cookei* tick has been endemic to Québec for many years, only 6 cases of Powassan encephalitis were reported between 2004 and 2014. The very low number of cases suggests that the risk to humans of acquiring Powassan encephalitis is limited in the province [22].

We found that all instars of *I*. *cookei* ticks are present in Québec, and were collected more frequently from April to September. In South Ontario, this period of activity is reported to end earlier in August, with the larva appearing in spring slightly later than nymphs and adults [30] as was the case in Québec in the present study. However, in Québec, a second abundance peak for larvae occurred in November. This could be explained by the high abundance of *I*. *cookei* ticks submitted in Quebec compared with the study data in Ontario, allowing a more detailed assessment of seasonality description, or is perhaps a genuine difference associated with ecological/climatological factors. It is interesting to note that the *I*. *cookei* adult activity period is different that of *I*. *scapularis* (greatest adult activity in spring and autumn [17, 31]) and this suggests that if a human gets bitten by an adult tick in the summer season, there is a high chance that the tick is not an *I*. *scapularis* but rather an *I*. *cookei* tick.

Birds disperse several species of ixodid ticks in Canada during spring migration when they translocate ticks from the United States, and Central and South America [32]. Migrating birds had a significant role in introduction and range expansion of *I*. *scapularis* ticks northward Canada [33] but this role is limited for *I*. *cookei* tick species because these tick rarely parasitize birds [32–34]. Local host movement is likely more important in driving local-scale tick dispersal [8]. Our findings provided evidence for a recent range expansion of *I*. *cookei* ticks. In part the geographic pattern of expansion matches that of *I*. *scapularis* (north-eastern expansion in the province [11, 17]), possible expansion to the north of the Saint-Lawrence River was also observed.

The American dog tick, *D*. *variabilis*, is so far, less abundant in Quebec as compared with Ontario and Maine, which neighbour Québec, where this tick is the second most abundant species after *I*. *scapularis* [18, 19]. This difference may be due to environmental factors as climate, habitat and density of hosts [35–37] but our data possibly underestimate the real abundance of this tick given the cessation of passive tick surveillance from animal origin since 2009 in the south of the province.

Most *D*. *variabilis* ticks submitted in passive surveillance were adults. A small number of immature *D*. *variabilis* were collected by passive surveillance suggesting that this species is established in Québec. Numbers of immature *I*. *scapularis* submitted in passive surveillance are also low when animals are the main source of ticks presumably because immatures are difficult to find on pets [38]. However, in addition to this possible explanation, while adult *D*. *variabilis* readily bite humans [39], larvae and nymphs rarely do [40]. Since *D*. *variabilis* ticks rarely parasitize birds [33, 34], the abundance of specimens collected from passive surveillance in Québec suggest likely established populations rather than migratory bird-dispersed adventitious ticks.

Mature *D*. *variabilis* ticks activity was highest in late spring and in summer with tick abundance peaking in June as reported in southwestern Nova Scotia [41]. Few specimens were submitted outside of this period (February and March) which probably reflects ticks imported from travel to warmer areas. This species is considered as a vector of *Rickettsia rickettsii*, the agent of RMSF and this bacterium has been isolated from *D*. *variabilis* collected in southwestern Ontario during 1965–1971 and in Nova Scotia during 1976–1980 [22]. However, more recent studies have failed to find *R*. *rickettsii* in *D*. *variabilis*, and *Dermacentor andersoni*, collected in various locations across Canada [42]. The number of RMSF cases reported in Canada is low but is not a notifiable disease in Canada and therefore, the true incidence of this infection is unknown.

The lone star tick, *A*. *americanum*, is increasingly recognized as a vector of pathogens of importance for human and animal health [43]. The low number of *A*. *americanum* ticks collected in passive surveillance suggests that, as in Ontario [18], the lone star tick is not yet established in Québec. The specimens found are more likely introduced by migratory birds or travellers and their pets. However, we suggest vigilance for populations of *A*. *americanum* becoming established in Québec given the reduced passive surveillance effort in the south of the Province region at the same time that studies are suggesting a northward shift in the distribution of *A*. *americanum* ticks throughout the Midwest and northeastern United States over the past 50 years [44, 45].

The brown dog tick, *R*. *sanguineus*, is the most widespread tick in the world and a vector of pathogens which affect domestic animals and occasionally, humans. An outbreak of RMSF in Arizona in 2004 was caused by infected *R*. *sanguineus*: all patients in the outbreak reported contact with tick-infested dogs, indicating that dogs may serve as important transport hosts by carrying infected ticks close to their owners [46, 47]. This species is more commonly found in warmer climates and associated with human habitations and domestic dogs in urban, suburban and rural environments. *R*. *sanguineus* ticks submitted in Québec passive surveillance program are probably transient populations carried into homes by dogs owned by travellers or imported by breeders. Consistent with their survival in homes and kennels, where ticks are protected from cold winter, specimens of this tick were submitted during all months of the year.

The numbers of the four most submitted tick species have increased between 2007 and 2015 in Québec at the same time that *I*. *scapularis* tick abundance has increased in this province associated with spread of the geographic range of reproducing populations [17]. It is possible that climate change is co-driving the range expansion of these ticks and their associated pathogens as suggested by studies elsewhere in North America [13, 14] and Europe [48]. For *I*. *cookei*, the most abundant tick species after *I*. *scapularis*, the number of ticks submitted in passive surveillance more than doubled from 2007 to 2015. Numbers of submissions of *D*. *variabilis*, *R*. *sanguineus* and *A*. *americanum* also increased during the same period. This is different from Ontario and Maine where only increases in numbers of submitted *I*. *scapularis* ticks (for 2008–2012 and 1989–2006 periods, respectively) have been reported. These differences may be explained by ecological and climatic factors or by the study periods. However, perhaps, since the emergence of Lyme disease in Québec, the awareness of the population toward tick bites has increased leading to more submission of ticks in the program.

Women were more often bitten by *I*. *cookei* ticks than men which is the opposite of what has been shown for *I*. *scapularis* ticks in Canada [18, 49, 50] and the United States [19, 51]. Following recent Lyme disease awareness campaigns women have been reported as more frequently avoiding known Lyme disease risk areas and avoiding risk behaviors [52–54]. So why women are more frequently bitten by *I*. *cookei* is unclear. Clearly though, *I*. *cookei* is a nidicolous tick associated mostly with the woodchucks, while *I*. *scapularis* which is exophilic and perhaps this behavioural difference results in different risk activities for exposure to bites of these tick species.

Children below 10 years of age and adults aged 55–69 years were most frequently bitten by *I*. *cookei* ticks. *Ixodes cookei* is the primary vector of Powassan Virus, a severe disease with possible neurological sequelae that most frequently affects children in North America [26]. In addition, children aged below 10 years were also bitten by *D*. *variabilis* the vector of RMSF, which is a potentially fatal disease [55]. The high exposure of children to tick bites whether from *I*. *cookei* and *D*. *variabilis* as shown in the present study, as well as their high risk exposure to *I*. *scapularis* [17–19, 49, 51] and the potential infection risk from pathogens associated with these ticks (Powassan virus, *R*. *rickettsii* and *B*. *burgdorferi*) underline the importance of continued surveillance. Active surveillance is the gold standard method for identifying where ticks and pathogens are in the environment. However, passive surveillance has the advantage of being able to measure the risk to humans. For example, active surveillance identified the presence of established populations of *H*. *leporispalustris* in Québec (data not shown). However, despite its widespread presence in the environment in the region studied, the passive surveillance data demonstrate there is little risk for humans posed by this tick as no human was bitten by this species during the study period.

## Conclusions

This study provides the first assessment of ticks of public health importance in Québec other than the Lyme disease vector *I*. *scapularis*.

Twelve tick species other than *I*. *scapularis* were submitted through the passive tick surveillance program. The most frequently submitted ticks were *I*. *cookei*, *D*. *variabilis*, *R*. *sanguineus* and *A*. *americanum*, and there was evidence that the first two have reproducing populations established in Quebec. There was evidence that the abundance and geographic range of *I*. *cookei* is increasing in the region, and we speculate that this may also be a consequence of the warming climate that has co-driven the expansion of the range of *I*. *scapularis*.

Demographic information showed that children were more bitten by *I*. *cookei* and *D*. *variabilis* ticks, representing a population group most at risk of tick-bite that need to be targeted by an awareness campaign targeting them directly or their parents. Women are more likely bitten by *I*. *cookei* ticks than men but further studies are needed to understand why.

The increasing risk of tick bites from multiple species highlights the need for entomological surveillance for medically important ticks and their associated pathogens in this region.
